# A Review and Secondary Analysis of Competition-Related Impacts of Nonindigenous Aquatic Plants in the Laurentian Great Lakes

**DOI:** 10.3390/plants10020406

**Published:** 2021-02-20

**Authors:** Rochelle Sturtevant, El Lower, Austin Bartos, Ashley Elgin

**Affiliations:** 1 Michigan Sea Grant, Michigan State University Extension, NOAA-GLERL, Ann Arbor, MI 48108, USA; 2 Michigan Sea Grant, University of Michigan, NOAA-GLERL, Ann Arbor, MI 48108, USA; Erika.Lower@noaa.gov (E.L.); Austin.Bartos@noaa.gov (A.B.); 3 NOAA Great Lakes Environmental Research Laboratory, Lake Michigan Field Station, Muskegon, MI 49441, USA; Ashley.elgin@noaa.gov

**Keywords:** nonindigenous species, aquatic, Great Lakes, competition

## Abstract

The Laurentian Great Lakes of North America are home to thousands of native fishes, invertebrates, plants, and other species that not only provide recreational and economic value to the region but also hold an important ecological value. However, there are also 55 nonindigenous species of aquatic plants that may be competing with native species and affecting this value. Here, we use a key regional database—the Great Lakes Aquatic Nonindigenous Species Information System (GLANSIS)—to describe the introduction of nonindigenous aquatic plants in the Great Lakes region and to examine patterns relating to their capacity to compete with native plants species. Specifically, we used an existing catalog of environmental impact assessments to qualitatively evaluate the potential for each nonindigenous plant species to outcompete native plant species for available resources. Despite an invasion record spanning nearly two centuries (1837–2020), a great deal remains unknown about the impact of competition by these species. Nonetheless, our synthesis of existing documentation reveals that many of these nonindigenous species have notable impacts on the native plant communities of the region in general and on species of concern in particular. Furthermore, we provide a thorough summary of the diverse adaptations that may contribute to giving these nonindigenous plants a competitive advantage. Adaptations that have been previously found to aid successful invasions were common in 98% of the nonindigenous aquatic plant species in the database.

## 1. Introduction

Freshwater ecosystems have been deeply transformed by invasive species [1,2]. Nonindigenous aquatic plants have been identified as a major cause of biodiversity loss in many countries [3,4]. Problems associated with the spread of nonindigenous aquatic plant species are increasing in Europe and the Mediterranean [5,6,7], Australia [8], South Africa [9], tropical inland waters worldwide [10], and North America [11,12,13]. However, there are few studies of the patterns in community structure and composition available for aquatic plants, as are common in terrestrial communities [14] or even for planktonic microflora [15].

The Laurentian Great Lakes are home to thousands of native fishes, invertebrates, plants, and other species that not only provide recreational and economic value to the region but also hold an important ecological value. With more than 180 aquatic nonindigenous species documented in the region, the Great Lakes basin is considered one of the most heavily invaded aquatic systems in the world [16,17,18]. Some of these nonindigenous species may become invasive (i.e., “those species whose introduction does or is likely to cause economic or environmental harm or harm to human health” [19]) and threaten the ecological and/or socioeconomic value of the Great Lakes.

Of these 180+ known Great Lakes nonindigenous species, 55 (29%) are plants (Table 1). Aquatic plants have been among the most neglected components in ecological studies of aquatic ecosystems [20] and the Great Lakes are no exception. However, aquatic plants play a major role in ecosystem services associated with the value we place on these systems. By providing food and habitat for fish and aquatic invertebrates, oxygenating the water, limiting erosion, and influencing nutrient levels, plants contribute extensively to the overall structure and function of aquatic environments [21]. Impairments to aquatic plants thus have the potential to negatively affect a wide range of services as well as ecosystems as a whole.

The impact of nonindigenous aquatic plants on native aquatic plants is examined here only in the context of impact through competition. Competition has long been recognized as a significant driver of community succession [23] and structure [24], which are critical components of impact, but studies of these interactions in aquatic plant communities remain rare [25]. Our operational definition of competition is quite broad, encompassing any negative interference of one plant over another in capturing resources, most notably space. We include direct and indirect negative impacts, ability to tolerate or avoid suppression, resource competition (for light, water, nutrients, etc.), and interference competition (such as allelopathy) [26,27]. We categorize competition primarily in broad qualitative outcome-oriented terms, (a) significant adverse effects (e.g., critical reduction, extinction, and behavioral changes) on one or more native species populations and (b) noticeable stress to or decline of at least one native species population, and only secondarily review and categorize potential mechanisms (species traits) identified in the database profiles.

The National Oceanic and Atmospheric Administration’s (NOAA) Great Lakes Aquatic Nonindigenous Species Information System (GLANSIS) serves as a regional information clearinghouse for science-based information supporting management of aquatic nonindigenous species in the Great Lakes. GLANSIS provides comprehensive bibliographies, systematic review, summary profiles of each species, distribution mapping, and impact assessment for each of the nonindigenous species overwintering and reproducing below the ordinary high water mark of the Great Lakes, including 55 nonindigenous species of aquatic plants. Here, we examine the subset of the database that addresses the competitive impacts of the nonindigenous aquatic plants. It is our aim that this review serves as a synthesis of regional information on the impacts of aquatic nonindigenous plants to native aquatic plants via competition, much of which is not otherwise readily available, a proof-of-concept for the value of inclusion of impact information (even qualitative) in invasive species databases, and highlights potentially important future research needs.

## 2. Results

**Figure 1:**
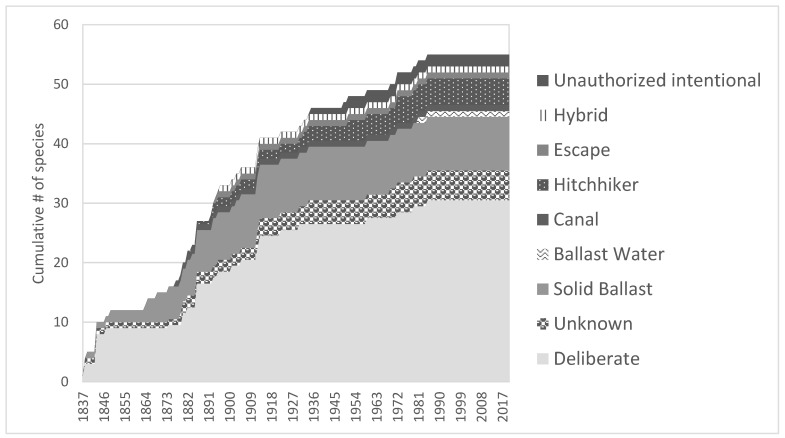
Cumulative introduction of nonindigenous plants to the Great Lakes basin by vector of introduction. Note the Hybrid vector represents recombination of a nonindigenous captive species introduced intentionally as a garden plant with a native plant, probably via cross-pollination.

The history of nonindigenous aquatic plants in the Great Lakes is long and differs markedly from the overall pattern of invasion for the Great Lakes [11]. Records show that the first nonindigenous aquatic plant (*Rumex obtusifolius*) was introduced to the Great Lakes basin around 1837 [28]. Forty aquatic plant species were introduced prior to 1920, while only 15 were introduced within the last century, with the peak rate of introduction occurring in the early 1880s (Figure 1). Fifty-five percent of the introductions of nonindigenous plants to the Great Lakes are attributed to deliberate introductions, while 18% came from shipping vectors (solid ballast and ballast water). This contrasts with the pattern for all nonindigenous introductions to the Great Lakes, which peaked in the late 1990s, with 20% of the introductions (including plants) associated with deliberate introductions and 40% associated with shipping [11]. Aquatic nonindigenous plants thus form a unique subset of the overall invasion of the Great Lakes.

**Figure 2:**
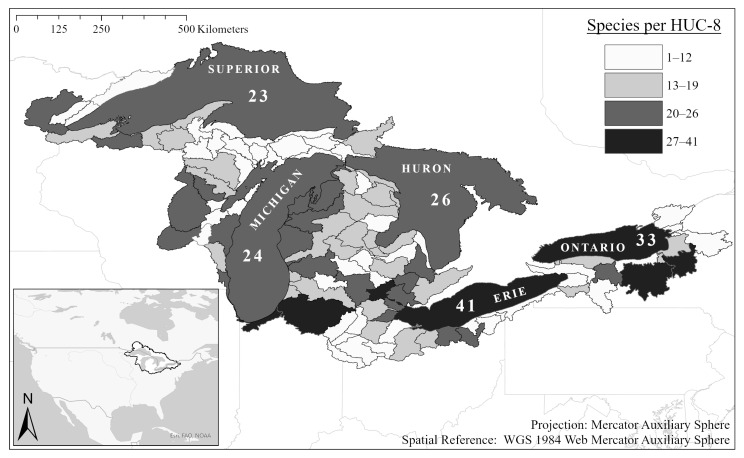
Distribution of aquatic nonindigenous plants in the Great Lakes basin by sub-watershed.

At present, most of these nonindigenous aquatic plant species are widespread throughout the Great Lakes basin, though not every species is found in every sub-watershed (Table 1 and Figure 2). However, a distinct pattern is apparent, wherein the lower lakes (Lake Erie and Lake Ontario)—which are both downstream and more southern—host more aquatic nonindigenous plant species per watershed than the upper lakes. It remains uncertain whether the lack of reports for the upper peninsula of Michigan (south of Lake Superior, with low human population density) represents a true lack of invasion or an underreporting of occurrences in these areas.

Just under half of the 55 nonindigenous plant species in the Laurentian Great Lakes have some level of reported competitive impacts on native plants. Most of these reports are observational and qualitative in the original sources, not quantitative. Five species (*Lythrum salicaria*, *Typha angustifolia*, *Iris pseudacorus*, *Frangula alnus*, and *Phragmites australis* ssp. *australis*) were found to have significant adverse effects as a result of competing with native plant species, and 19 were found to have resulted in some noticeable stress to or decline of at least one native plant population attributed to competition. Sufficient evidence concludes that 11 species have had no significant competition-based impacts to native plants in the Great Lakes basin (but may have had other significant impacts not related to competition).

**Figure 3:**
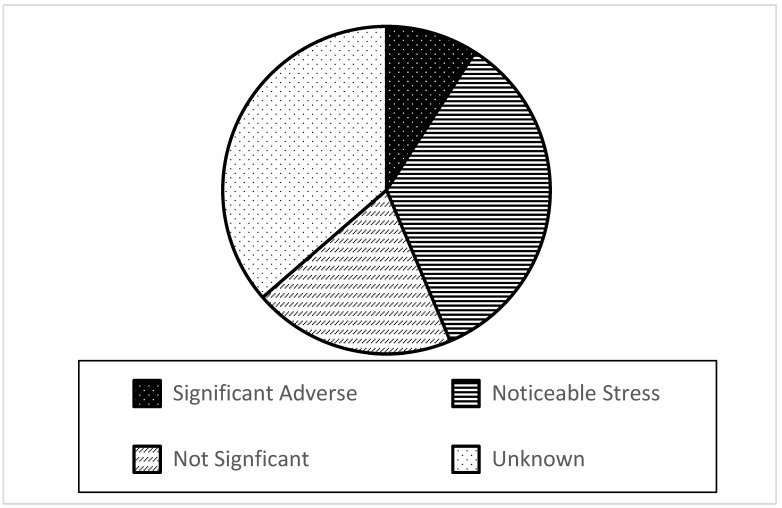
Nonindigenous aquatic plants by impact category—limited to impact attributed to competition with native plant species (n = 55). (Re-analysis of the impact assessments which appear in [29,30,31,32,33,34,35,36,37,38,39,40,41,42,43,44].

For the remaining 20 species (35%), too little information was available to draw conclusions about whether competitive impacts occur in the Great Lakes (Figure 3). These unknowns occur either because there is a lack of relevant literature or because sources disagree on the impact. Review of the literature for each of the 55 nonindigenous aquatic plants in the Great Lakes region revealed an average of just 3.5 citations per species relevant to the question of impacts of these species via competition (range 0 to 16). Cited studies contributing to “unknown” classification include those with a preponderance of published expert opinion (e.g., in the discussion or conclusion of a published paper) rather than reliance on direct laboratory or field evidence of competition.

There are notable effects of competition with nonindigenous plant species that impact native plant communities. In our review, we found forty-six native species documented to experience, or speculated as likely to experience, direct competition from 22 nonindigenous plant species (Table 2). We found most of these identified impacted native plant species are congeneric with the invader harming them (80 of 91 pairwise competitive interactions identified at the species level are congeneric) and/or have been identified as having a high degree of niche overlap. From our data collection, we cannot speculate whether congeneric pairs experience greater competition or whether this subset (congeneric interactions) has merely been studied more frequently. This list of impacted species includes numerous state/provincially designated at-risk species. Note that status of these at-risk species varies considerably among jurisdictions within the larger region. This list of impacted at-risk species includes the following:

- extirpated (*Chenopodium capitatum* (OH = Ohio), *Chenopodium leptophyllum* (OH), *Juncus greenei* (PA = Pennsylvania), and *Juncus militaris* (IN = Indiana));
- endangered (*Chenopodium foggii* (PA), *Echinochloa walteri* (PA), *Epilobium angustifolium* (IN and OH) *Lysimachia radicans* (IL = Illinois), *Epilobium strictum* (PA), *Iris brevicaulis* (OH), *Iris cristata* (PA), *Iris verna* (PA), *Iris virginica var. shervei* (NY = New York and PA), *Rhamnus alnifolia* (IL), *Rhamnus lanceolata* (PA), *Myriophyllum sibericum* (PA), *Juncus alpinus* (IL), *Juncus ambiguous* (NY), *Juncus biflorus* (NY), *Juncus brachycephalus* (NY and PA), *Juncus dichotomus* (OH and PA), *Juncus diffusissimus* (OH), *Juncus ensifolius* (NY), *Juncus greenei* (OH), *Juncus interior* (OH), *Juncus militaris* (PA), *Juncus scirpoides* (NY and PA), *Juncus secundus* (IN), *Juncus stygius* (WI = Wisconsin), *Juncus subcaudatus* (NY), *Juncus vaseyi* (IL), *Lycopus amplectens* (IN), and *Lycopus rubellus* (NY and PA));
- threatened (*Chenopodium subglabrum* (CAN = Canada), *Cirsium pitcheri* (US = United States, IL, IN, MI = Michigan, WI, and ONT = Ontario), *Epilobium strictum* (IL and OH), *Iris lacustris* (US, MI, and WI), *Iris verna* (OH), *Myriophyllum sibiricum* (OH), *Juncus alpinus* (PA), *Juncus biflorus* (PA), *Juncus brachycephalus* (MI), *Juncus militaris* (MI), *Juncus pelocarpus* (IN), *Juncus scirpoides* (IN and MI), *Juncus secundus* (OH), *Juncus stygius* (MI), *Juncus vaseyi* (MI), and *Lycopus virginicus* (MI));
- special concern (*Cirsium pitcheri* (CAN), *Iris lacustris* (CAN and ONT));
- vulnerable (*Ilex verticillata* (NY)); and
- endemic (*Cirsium pitcheri*, *Iris lacustris*, and *Iris robusta*) species in the Great Lakes region.

**Figure 4:**
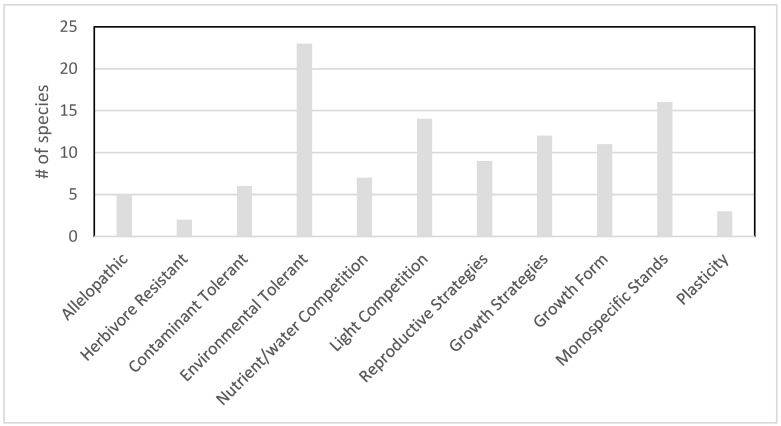
Adaptations identified as assisting in competition (n = 55; however, species can have multiple adaptations). Species scoring “unknown” or “low impact” typically also have adaptations noted and are included in this analysis.

Competition between plants is driven by the traits that allow plants to capture resources and to monopolize space. Understanding the mechanisms that enable plants to compete for limiting resources can foster better predictions regarding competitive outcomes and the consequences of competition [26]. Our review of the literature (including literature from the Great Lakes region, from other regions where the plant is invasive, and from the native ranges) reveals that aquatic plants nonnative to the Great Lakes region have a diverse array of adaptations that may help them outcompete native species (Figure 4). Adaptations that have been previously found to aid successful invasions were common in 98% of plant species in the database. Many of these are common adaptations of wetland plants in fluctuating environments and may also be characteristics of some or even many Great Lakes native species; thus, we provide this list of adaptations only as a starting point for future investigations into the mechanisms behind competition. It is possible that there are many more cases of adaptations by nonindigenous plants conferring a competitive advantage that have yet to be documented due to a lack of research. Even within our very broad and qualitative categories, there is significant diversity of adaptations. Many species have multiple adaptations, with an average of 1.9 adaptation categories per species (range 0 to 6). We do not attempt to discuss the relative significance of particular adaptations within a species or across the suite because the adaptations were most often noted in distinct habitats and studied using different methods, which impedes meaningful comparison. The general principles of invasion mechanisms are unlikely to hold up across ecosystem types, but it is worth searching for general patterns within distinct ecosystem units [13].

Five species (*Nitellopsis obtusa*, *Lupinus polyphyllus*, *Iris pseudacorus*, *Salix caprea*, and *Solanum dulcamara*) are identified as allelopathic, producing chemicals that inhibit germination, growth, or reproduction of potential competitors [48,49,50,51,52,53]. Two additional species (*Chenopodium glaucum* and *Conium maculatum)* are identified as producing chemicals (saponins and piperidine alkaloids, respectively) that inhibit herbivory (included in Figure 4 as Herbivore Resistant), which increases their ability to outcompete plants that suffer significant herbivore damage [54,55,56,57,58,59].

Contaminant tolerance, defined here as tolerance to chemicals or elements that are anthropogenic in origin and not a natural component of Great Lakes habitats, is cited as an adaptation providing a competitive advantage to nonindigenous aquatic plants for six of the species. *Conium maculatum* is tolerant to heavy metal contamination and is frequently found growing on old industrial sites in the Great Lakes region, where this tolerance may be a significant factor in its ability to outcompete native species [60]. The other five species (*Pluchea odorata*, *Solidago sempervirens*, *Alopecurus geniculatus*, *Puccinellia distans*, and *Typha angustifolia*) are all identified as salt-tolerant [28,33,61,62,63,64,65,66,67,68]; all of these except *Typha angustifolia* are limited in their distributions and found primarily in proximity to sites with significant salt contamination.

Broad “environmental tolerances” are frequently cited as a characteristic of successful invasive species [69,70,71]. We define this category very broadly and qualitatively, including both species that have broad tolerances as well as those with particular tolerances or tolerances to fluctuations. These tolerance include those falling within the natural range of Great Lakes variability (e.g., seasonal temperature and water level fluctuations) as well as even broader ranges, which are anthropogenically influenced (e.g., eutrophication/anoxia due to nutrient pollution, management manipulation of water levels, etc.). Beyond any direct competitive advantage, these adaptations may also aid nonindigenous species in establishing either through expanding habitat suitability (either broadly or to particular vulnerable microhabitats) or by increasing propagule pressure (decreasing propagule loss in transit). Twenty-three nonindigenous plant species fall into the category of “environmentally tolerant”. *Conium maculatum* tolerates high nitrogen conditions [72]. *Najas minor* is more tolerant to turbidity and eutrophic conditions than native *Najas* spp. [73]. *Butomus umbellatus*, *Cabomba caroliniana*, *Epilobium hirsutum*, and *Lysimachia vulgaris* tolerate water level fluctuations (common in Great Lakes coastal wetlands) [74,75,76,77,78,79,80,81], while *Myosoton aquaticum* and *Veronica beccabunga* are tolerant to drying and will survive management drawdowns [82,83,84]. *Cabomba caroliniana*, *Persicaria maculosa*, and *Agrostis gigantea* tolerate acidic soils [75,85,86], while *Salix alba* is described as “pH adaptable” or tolerant of both acidic and alkaline conditions [87]. *Iris pseudacorus* and *Alopecurus geniculatus* are adapted to low oxygen [33,64,88,89].

Seven species are noted as having adaptations that make them superior competitors for nutrients. *Nasturtium officinale* is a superior competitor for nitrate [90], *Carex acutiformis* is noted for highly efficient nitrogen use [91,92], and *Alnus glutinosa* roots bear nodules with bacteria (*Frankia alni*) that assimilate atmospheric nitrogen [93,94].

Fourteen species are noted as having adaptations that help them be superior competitors for light. Again, this category is defined broadly and qualitatively to include both positioning (higher in the water column allowing shading), seasonal (e.g., early spring growth shading competitors, extended growing seasons, etc.), and production of litter (which shades competitors seeds) as well as direct physiological differences in photosynthetic efficiency (leaf size, angle, and shade tolerance). *Cabomba caroliniana*, *Nitellopsis obtusa*, *Hydrocharis morsus-ranae*, *Najas minor*, *Trapa natans*, and *Nymphoides peltata* are reported to form mats, which shade out the species below them [42,95,96,97,98,99,100,101,102,103,104,105]. *Frangula alnus* is shade-tolerant [106,107,108], indirectly functioning to extend its growing season and potentially the period during which it shades competitors (and competitors’ seedlings). *Salix fragilis*, *Salix purpurea*, and *Conium maculatum* shade the understory preventing growth of competitors that require high light levels [55,109,110]. *Typha angustifolia* produces a heavy litter that shades soils to prevent the emergence of competitors [43]. *Marsilea quadrifolia* plants are able to adjust the angle of their floating leaflets to optimize access to sunlight and their ability to photosynthesize [111], which may promote early growth and/or shade submergent competitors.

Reproductive strategies are noted as aiding in competition and as common characteristics of successful invasive species [112]; nine species examined here are identified as having adaptations in this category. Here again, we defined the category broadly and qualitatively, including adaptations relating to pollination, seed set, dispersal, seed banks, and seed viability. *Chenopodium glaucum* and *Echinochloa crus-galli* are self-pollinators [113,114]. *Impatiens glandulifera* and *Iris pseudacorus* are considered superior competitors for the attention of pollinators [115,116,117,118]. *Cirsium palustre* is a monocarpic perennial that can delay seed-set until conditions are ideal [119,120]. Long-distance spread of seeds with relatively high viability is noted as improving competition for *Najas marina* [121], extremely high seed-set and long-lived seed influences competition in favor of *Lythrum salicaria* [122], and dominance of viable seeds in the seed bank is also a factor for *Juncus inflexus* [123]. Similarly, twigs of *Salix fragilis* can float downstream and take root, which allows the species to spread rapidly and influences its ability to outcompete natives [124].

Growth strategies potentially aiding competition were also defined broadly and qualitatively but were limited to those likely to directly influence competition for space through seasonal growth strategies or longevity. Twelve species have documented growth strategies that aid competition. *Myriophyllum spicatum*, *Hydrocharis morsus-ranae*, *Glyceria maxima*, and *Potamogeton crispus* are noted in the literature and/or state agency reports to begin growth in early spring, getting a head start on competitors [96,125,126,127,128,129,130,131,132,133]; *Myriophyllum spicatum* may overwinter on the bottom (winter-green), and Potamogeton crispus is noted to germinate in the fall and to overwinter. *Nitellopsis obtusa* is noted as able to grow in the late fall, supporting a more robust overwintering population [134]. *Rorippa sylvestris*, *Carex disticha*, *Echinochloa crus-galli*, *Lycopus asper*, *Lycopus europaeus*, *Persicaria maculosa*, and *Salix caprea* are pioneer species [28,53,135,136,137,138,139,140,141] and are quick to invade disturbed sites, with a rapid growth rate that outpaces competitors. *Carex disticha* is long-lived relative to other pioneer species, which helps it to persist past the early successional stage [136].

The formation of dense mats or rhizomes (growth form) was treated as a separate category from growth strategy (seasonal timing). Closely related, the documentation of monospecific or monoclonal stands was also considered but eventually treated as a separate category. Sixteen species are documented to form monospecific stands, and eleven are reported to form mats or to spread by dense rhizomes (designated “Growth form” in Figure 4). Although similar (3 species appear on both lists), not all those forming mats have been documented to form monospecific stands and not all those that form monospecific stands do so by means of dense clonal rhizome/root networks. *Juncus inflexus* (monospecific tufts from the joint root system [142]), *Marsilea quadrifolia* (rhizomes [143,144]), and *Glyceria maxima* (rhizomes [145,146,147]) form monospecific stands by means of dense underground networks that exclude competitors. *Cirsium palustre*, *Impatiens glandulifera*, *Alnus glutinosa*, *Myosotis scorpioides*, *Cabomba caroliniana*, *Carex acutiformis*, *Myriophyllum spicatum*, *Iris pseudacorus*, *Epilobium hirsutum*, *Veronica beccabunga*, *Phragmites australis* ssp. *australis*, *Rumex longifolius*, and *Typha angustifolia* have also been documented to form monospecific stands but may rely on other mechanisms to do so [45,68,95,100,105,120,148,149,150,151,152,153,154,155,156,157]. While *Hydrocharis morsus-ranae*, *Juncus compressus*, *Mentha aquatica*, *Mentha spicata*, *Mentha x gracilis*, *Agrostis gigantea*, *Lysimachia nummularia*, and *Solanum dulcamara* can form rhizome or dense root mats [79,80,101,102,132,146,158,159,160,161,162,163], we have not found documentation of these species forming sizeable monospecific stands.

Variability due to either phenotypic plasticity or genetic strain diversity is documented as a contributing factor to competition for three nonindigenous aquatic plant species of the Great Lakes region. Genetic variability in nonindigenous populations of *Impatiens glandulifera* have been documented to contribute to its ability to adapt to local environments within a few generations [164]. *Frangula alnus* demonstrates a high degree of phenotypic plasticity in bud burst timing [165]. *Solanum dulcamara* shows phenotypic plasticity with regard to height, leaf morphology, and chlorophyll production in accordance with environmental conditions (light, temperature, and water) [166,167,168,169].

## 3. Discussion

Aquatic nonindigenous plants form a unique subset of the invasion history of the Great Lakes. Despite an invasion record spanning nearly two centuries (1837–2020), a great deal remains unknown about these species; for 35% of the species, the impact to native plants via competition remains unknown at even the coarsest level (significant/noted/none documented). Additional data collection is needed for all nonindigenous aquatic plant species to determine, refine, and quantify competitive impacts.

Nonetheless, many of these nonindigenous species have notable impacts to the native plant communities of the region. At least 46 native species are identified as significantly impacted by competition from these invasive species, including many species designated legally as of special concern (endangered, threatened, etc.). These numbers likely do not adequately capture the true magnitude of competitive impact as many additional studies highlight impact to native assemblages (e.g., “high marsh community” and “sedges”) without calling out the individual species impacted. Likewise, many studies of native communities note that they are “impacted by competition from invasives” without specifying which invasive species are responsible. These impacts are widespread; sub-watersheds of the region average 14 nonindigenous aquatic plant species each, and none remain untouched by nonindigenous aquatic plants. The five invasive aquatic plants falling in our top tier for “significant adverse effects” due to competition (documented as responsible for critical reduction, extinction, or behavioral changes to one or more native species populations) include *Lythrum salicaria*, *Typha angustifolia*, *Iris pseudacorus*, *Frangula alnus*, and *Phragmites australis* ssp. *australis.* All five are widespread emergent wetland plants first introduced to the region more than a century ago. Each of these species have at least two documented adaptations that contribute to their being superior competitors over the native plants, and collectively, they cover nearly the entire array of adaptation categories.

Adaptations categorized as environmental tolerances (23 species), contaminant tolerances (6 species), nutrient competition (7 species), and light competition (14 species) all likely serve to help species gain a competitive advantage in particular microhabitats or under fluctuating conditions. Many aquatic nonindigenous plant species have specific tolerances that help them to outcompete natives in particular habitat types, such as sites contaminated with heavy metals, salt, or nitrogen or sites experiencing eutrophication, turbidity, or acidification. These tolerances likely contribute to these nonindigenous species, gaining a foothold in disturbed sites. Some appear to remain restricted to these marginal habitats, while others spread to more pristine habitats. This finding supports the general hypothesis that aquatic plant species differ in their responses to sediment composition and irradiance [170], with natural distributions of plant species correlated to these abiotic factors. Although the authors concluded that interspecific competition played only a minor role in structuring native submerged plant communities [25], they allowed the possibility that interspecific competition could occur during establishment phase, which would be a more critical consideration for interactions between nonindigenous and native plants in comparison to the interactions among native species that they were studying.

The prevalence of salt-tolerant species (five nonindigenous aquatic plants in this analysis) among the invaders of freshwaters is an interesting case that may be worthy of additional study. Salt contamination (largely from road salt) is a significant issue for many historically freshwater wetlands and marginal habitats (such as roadside ditches) in the region [171,172,173], and salt contamination potentially influences the success of these species in competition with natives that are not adapted to salt.

Other nonindigenous aquatic plants species noted in the literature as “broadly tolerant” or tolerant of a range of soil, nutrient, water, and other conditions are able to spread more broadly in part because of these tolerances and, thus, are competitive in a broader array of habitats, in disturbed habitats, and/or in habitats where conditions fluctuate. While not tidal, water level fluctuations at both short and long temporal scales are a common feature of the Great Lakes nearshore. Storm surge and subsequent seiches in the Great Lakes can cause water levels to change in excess of 2 m in under an hour. Annual cycles typically place average summer high water levels a half-meter or more above the winter low, and longer term fluctuations (climate signals) are also apparent in the historic record (https://www.glerl.noaa.gov/data/dashboard/portal.html (accessed on 15 December 2020)). For inland waters and managed wetlands of the region, water level control (drawdowns and/or flooding) is often used as a management strategy for controlling nonindigenous species (both plants and fish). Broadly tolerant species, particularly those with respect to water depth, may be at a competitive advantage in these fluctuating systems.

Plant species not called out in this particular analysis should not be assumed to have no impact. This paper focuses on summarizing only a single component of risk: the impact of nonindigenous aquatic plants to native aquatic plants via competition. Other potentially important components of impact (which are also summarized in GLANSIS products) were excluded from this analysis in an effort to focus on competition alone. For one example, a recent ranking of the highest impact invasive species in the Great Lakes [174] included only one aquatic nonindigenous plant, *Myriophyllum spicatum* (Eurasian watermilfoil), which is not among the top impact species when we consider only competition. Eurasian watermilfoil had only a moderate impact score for competition, meaning that its successful establishment has resulted in some noticeable stress or decline in native populations due to its ability to grow in dense stands, allowing it to successfully outcompete other native species for nutrients, sunlight, and space and eventually resulting in a reduction in biodiversity of native plants [128,175]. However, in the broader analysis of diverse and indirect impacts, Eurasian watermilfoil scored higher due to many other documented types of impact (not related to competition [28,105,128,152,175,176,177,178,179]).

## 4. Materials and Methods

We performed the analysis using information found in the Great Lakes Aquatic Nonindigenous Information System (GLANSIS in Supplementary Materials, https://www.glerl.noaa.gov/glansis/ NOAA Great Lakes Environmental Research Laboratory, Ann Arbor, MI, USA; accessed on 1 September 2020). GLANSIS provides information about aquatic nonindigenous species in the Laurentian Great Lakes region of North America and imposes the following criteria for inclusion of nonindigenous aquatic plants in the database:

- Geographic criterion: Only species that are found in the Great Lakes basin below the ordinary high water mark—including connecting channels, wetlands, and waters ordinarily attached to the Lakes—were included in the GLANSIS nonindigenous species list. Species that have been collected from inland lakes within the Great Lakes basin but do not meet this geographic criterion were excluded.
- Aquatic criterion: The United States Department of Agriculture (USDA) wetland indicator status was used as a guideline for determining whether wetland plants should be included in the list. Obligate, facultative wetland, and facultative plants were included as aquatic. Facultative upland and upland plants were excluded, even if found below the ordinary high water mark.
- Nonindigenous criterion: The species included in the GLANSIS nonindigenous list were those that met at least three of the following criteria based on Ricciardi 2006 [18]:
  ⚬ The species appeared suddenly and had not been recorded in the basin previously.
  ⚬ It subsequently spreads within the basin.
  ⚬ Its distribution in the basin is restricted compared with native species.
  ⚬ Its global distribution is anomalously disjunct (containing widely scattered and isolated population).
  ⚬ Its global distribution is associated with human vectors of dispersal.
  ⚬ The basin is isolated from regions possessing the most genetically and morphologically similar species.
- Reproducing and overwintering criterion: A nonindigenous species was considered to be in at least the early stages of establishment if it had a reproducing population within the basin and was capable of overwintering, as inferred from multiple discoveries of adult and juvenile life stages over at least two consecutive years. Given that successful establishment may require multiple introductions, species were excluded if their records of discoveries were based on only one or a few non-reproducing individuals whose occurrence may reflect merely transient species or unsuccessful invasions.

GLANSIS follows taxonomic nomenclature as identified in the Integrated Taxonomic Information System (https://www.itis.gov/ as checked for this publication on 1 December 2020), with records for both nonnative and native species tied to ITIS Taxonomic Serial Numbers.

*Phalaris arundinacea*, which otherwise fits these criteria, was removed from the GLANSIS nonindigenous list and from this analysis due to recent controversy with regard to its native status. This species has both native and introduced populations in close proximity, since it is both native to North America and has had European transplants cultivated for agricultural use [180]. In general, *Phalaris arundinacea* was treated as a native species in North America [180] and in the Great Lakes region, with gene influence from nonindigenous populations [181,182,183,184,185].

*Nitellopsis obtusa*, starry stonewort, was the only nonindigenous macroalgae included in the GLANSIS database. This species has more in common with the nonindigenous aquatic plants than it does with the 26 species of planktonic algae also meeting listing criteria and included in the GLANSIS database. Thus, we chose to include starry stonewort in this analysis.

*Phragmites australis* ssp. *australis* was included in the GLANSIS database as a nonnative taxa meeting criteria above and, therefore, was also included in this analysis. However, it is worth noting that *Phragmites australis* ssp. *americanus* is native to the Great Lakes region. Records and information for *Phragmites australis* is included only to the extent that it is identified to the subspecies level; this effectively excludes a large body of literature and records that fail to make identification to the subspecies level.

The GLANSIS database carefully documents the historic distributions of each included species within the Great Lakes basin. Original data are drawn from the peer-reviewed literature, from state agency reports, from museum and herbaria collections, from data-sharing arrangements (limited to verified data) with other state and regional databases, and from other forms of verified reports. GLANSIS relies heavily on data-sharing arrangements with other platforms to compile these verified reports as well as accepts direct reports into the system but does not accept unverified direct reports from the general public or other databases (unless also verified by GLANSIS or United States Geological Survey staff). Overall, the GLANSIS database includes more than 40,000 point mapped collection records for nonindigenous aquatic plants in the basin, ranging from only 2 verified records for *Salix caprea* to 6189 verified records for *Lythrum salicaria*. Each species profile includes an interactive point map that allows public access to the original report source. The original source for each record is available through GLANSIS (link below the map for each species). Distribution data can also be downloaded as Geographic Information System layers via the GLANSIS Map Explorer interface. The map presented above (Figure 2) aggregates these data to the watershed scale (USGS Hydrologic Unit Code Level 8 cataloguing units, as outlined here: https://water.usgs.gov/GIS/huc.html as valid for 1 December 2020).

Staff affiliated with GLANSIS have conducted comprehensive impact assessments for the overwintering and reproducing aquatic nonindigenous species of the Great Lakes since approximately 2010 using a protocol detailed in NOAA Tech Memo 161 [29]. All relevant original literature (excluding duplicates) are entered into the database and available through the USGS Nonindigenous Aquatic Species (NAS) reference database (https://nas.er.usgs.gov/queries/references/default.aspx accessed on 15 January 2021) as well as linked from each GLANSIS profile; the total literature included in the NAS database for the 55 aquatic nonindigenous plant species included in this paper exceeds 3500 original sources. A subset of this literature (639 original sources for aquatic nonindigenous plants) was used in the development of impact assessments. Most sources are from peer-reviewed literature and/or government reports and include direct laboratory and field observations of competitive impacts as well as observations of decline in native plant species concomitant with expansion of the nonindigenous species. Statements appearing in the peer-reviewed literature that constitute expert opinion (e.g., statements in discussion and conclusion sections) are included but flagged in the assessments in a way that influences final categorization toward “unknown”. Each assessment is reviewed and updated at least every fifth year based on a review of new literature and subject to external review. Full results of these impact assessments for each species, including all original references used in the development of both the full assessment and the particular subset examined here, are available in the Appendices of TM-161, TM-161b, and TM-161c [29,30,31]. Additional unpublished impact data may in some cases be attached to each collection record; while nominally accessible individually via the database, these are not easily extracted as a whole set from the current portal and are largely excluded from this analysis unless also noted in the Technical Memo series. Current summaries for each species (and critical literature citations) are also available online at https://www.glerl.noaa.gov/glansis (accessed on 15 January 2021).

The core current secondary analysis presented here is based on a single qualitative component from the larger assessment. Under the category of environmental impact, we assessed the question “Does it [the particular species assessed] outcompete native species for available resources (e.g., habitat, food, nutrients, light)?” Each species is noted as either of the following:

- yes, and it has resulted in significant adverse effects (e.g., critical reduction, extinction, and behavioral changes) on one or more native species populations;
- yes, and it has caused some noticeable stress to or decline of at least one native species population;
- not significantly; or
- unknown.

Each assessment was independently sent for external review, and the expert opinion of the reviewers may potentially have influenced the final categorization.

GLANSIS includes a profile for each nonindigenous species present in the Great Lakes. These are reviewed at least every fifth year (alongside the assessments) and updated as needed. All original profiles and major updates are subjected to external expert review. These species profiles include a segment on means of introduction, which summarizes the vectors involved in the movement of the species to the Great Lakes. This documentation is used in the vector analysis presented here. These species profiles also include segments on identification, ecology, and management, which includes information on growth habits, habitat, physiological requirements and tolerances, food web interactions (including possible herbivore resistance), life history, fecundity, and control methods (including notes on herbicide resistance). All information used for the assessment of nonindigenous plant adaptations favoring competition over native plants was drawn from these profiles and/or the risk assessments. The profiles also include citations of the original studies from which all information is compiled.

## 5. Conclusions

The 55 aquatic nonindigenous plant species of the Laurentian Great Lakes form a unique subset of the overall invasion of this important freshwater resource. At least 46 native plant species in the Great Lakes—including many designated by local jurisdictions as endangered, threatened, or otherwise at risk—are significantly impacted by competition from nonindigenous aquatic plants. The adaptations that give these nonindigenous species a competitive advantage over native species are diverse.

This analysis of the impact of nonindigenous aquatic plants to native plants of the Laurentian Great Lakes via competition highlights several broad areas in which further research is needed.

- For 35% of the aquatic nonindigenous plants species in the Great Lakes, it is unknown whether they have an outcome-based competitive impact on native plants. Further study of potential competitive impacts is needed for each of these 20 species.
- While for many nonindigenous aquatic plant species extensive information is available on species traits that may contribute to giving the nonindigenous species a competitive advantage over native species, a direct mechanistic-based examination of the competition between these species is generally lacking and is an area ripe for future research.
- For some nonindigenous aquatic plant species, competitive impacts to native species are documented only at the group level (e.g., impact to native sedges). More work is needed to understand the impacts on particular native plant species within these broader groups, which may include additional at risk natives.
- Competition is only one interaction through which nonindigenous aquatic plants can impact the ecology and economy of the Great Lakes region. More research is needed to place competitive impacts in the context of the broader suite of impact mechanisms.
- The potential alteration of several adaptations (e.g., environmental tolerance and light competition) as a result of climate change should be studied to properly understand how the nature of competition between nonindigenous and native plants may change under various climate scenarios.

## Figures and Tables

**Table 1:** Great Lakes nonindigenous aquatic plants included in the Great Lakes Aquatic Nonindigenous Species Information System (GLANSIS). Total plant species included = 55 (of 188 total nonindigenous species). The date range for all records in the database spans 1709–2019, with the first plant introduction dated to 1837. Total hydrologic units (HUC8s) in the basin = 105. Source: [22]. * noticeable stress, ** significant adverse effect.

Family	Nonindigenous Plant Species	Growth Form	Earliest Date	Inhabited HUC8s
Amaranthaceae	*Chenopodium glaucum*	Emergent	1865	38%
Apiaceae	*Conium maculatum* *	Emergent	1843	33%
Asteraceae	*Cirsium palustre* *	Emergent	1934	36%
	*Pluchea odorata*	Emergent	1912	3%
	*Solidago sempervirens*	Emergent	1969	6%
Balsaminaceae	*Impatiens glandulifera*	Emergent	1912	10%
Betulaceae	*Alnus glutinosa* *	Emergent (tree)	1913	15%
Boraginaceae	*Myosotis scorpiodes*	Emergent	1886	50%
Brassicaceae	*Nasturtium officinale*	Floating/Prostrate	1847	37%
	*Rorippa sylvestris*	Emergent	1884	25%
Butomaceae	*Butomus umbellatus* *	Emergent	1905	43%
Cabombaceae	*Cabomba caroliniana* *	Submerged w/Emergent flowers	1935	12%
Caryophyllaceae	*Myosoton aquaticum*	Emergent	1894	10%
Characeae	*Nitellopsis obtuse* *	Submerged	1981	36%
Cyperaceae	*Carex acutiformis* *	Emergent	1951	3%
	*Carex disticha*	Emergent	1972	1%
Fabaceae	*Lupinus polyphyllus*	Emergent	1959	14%
Haloragaceae	*Myriophyllum spicatum* *	Submerged	1880	87%
Hydrocharitaceae	*Hydrocharis morsus-ranae* *	Floating	1972	27%
	*Najas marina*	Submerged	1864	12%
	*Najas minor* *	Submerged	1932	28%
Iridaceae	*Iris pseudacorus* **	Emergent	1886	56%
Juncaceae	*Juncus compressus* *	Emergent	1895	18%
	*Juncus inflexus* *	Emergent	1922	5%
Lamiaceae	*Lycopus asper*	Emergent	1892	13%
	*Lycopus europaeus*	Emergent	1903	9%
	*Mentha aquatica*	Emergent	1843	25%
	*Mentha spicata*	Emergent	1843	26%
	*Mentha* × *gracilis*	Emergent	1896	10%
Lythraceae	*Lythrum salicaria* **	Emergent	1839	91%
	Trapa natans*	Floating	1949	9%
Marsileaceae	*Marsilea quadrifolia*	Floating/Emergent	1893	6%
Menyanthaceae	*Nymphoides peltata* *	Floating	1930	12%
Onagraceae	*Epilobium hirsutum* *	Emergent	1874	34%
Plantaginaceae	*Veronica beccabunga*	Emergent	1849	10%
Poaceae	*Agrostis gigantea*	Emergent	1838	70%
	*Alopecurus geniculatus*	Emergent	1882	9%
	*Echinochloa crus-galli*	Emergent	1838	51%
	*Glyceria maxima* *	Emergent	1979	8%
	*Phragmites australis* ssp. *Australis* **	Emergent	1869	33%
	*Poa trivialis*	Emergent	1843	12%
	*Puccinellia distans*	Emergent	1893	13%
Polygonaceae	*Persicaria maculosa*	Emergent	1838	41%
	*Rumex longifolius*	Emergent	1901	6%
	*Rumex obtusifolius*	Emergent	1837	36%
Potamogetonaceae	*Potamogeton crispus* *	Submerged	1879	75%
Primulaceae	*Lysimachia nummularia* *	Emergent	1882	38%
	*Lysimachia vulgaris* *	Emergent	1912	11%
Rhamnaceae	*Frangula alnus* **	Emergent (tree)	1913	56%
Salicaceae	*Salix alba*	Emergent (tree)	1886	23%
	*Salix caprea*	Emergent (tree)	1985	1%
	*Salix fragilis*	Emergent (tree)	1886	25%
	*Salix purpurea*	Emergent (tree)	1880	13%
Solanaceae	*Solanum dulcamara*	Emergent	1843	66%
Typhaceae	*Typha angustifolia* **	Emergent	1877	82%

**Table 2:** Identified pairwise interactions among nonindigenous and impacted native species. Nonindigenous species causing * noticeable stress and ** significant adverse effect; those without a symbol include both those categorized as low and unknown impact (per Figure 3); + at-risk native species; ^ terms per original source, exact species impacted were not provided in the original source. A high marsh community is defined as the intermittent zone between low marsh and uplands characterized by sandy soil. Understory natives refer to a reported displacement of plant species except for trees/shrubs. sources: [31,32,33,34,35,36,37,38,39,40,41,42,43,44,45,46,47].

Growth Form	Nonindigenous Plant Species	Impacted Natives
Submerged	*Myriophyllum spicatum* *	*M. sibiricum+*
Floating	*Trapa natans* *	Native (emergent) grasses
Emergent	*Chenopodium glaucum*	*C. album*, *C. berlandieri*, *C. capitatum +*, *C. foggii +*, *C. humile*, *C. leptophyllum +. C. overi*, *C. pallescens*, *C. pratericola*, *C. rubrum*, *C. salinum*, *C. simplex*, *C. standleyanum*, *C. subglabrum +*
Emergent	*Conium maculatum* *	Grasses and forbs
Emergent	*Cirsium palustre* *	*C. pitcheri+*, *C. muticum* *Carex* spp.
Emergent	*Pluchea odorata*	High marsh community ^
Emergent	*Butomus umbellatus* *	*Phalaris arundinacea*, *Phragmites australis* ssp. *americanus*, Non-specific natives
Emergent	*Carex acutiformis* *	Understory natives ^
Emergent	*Iris pseudacorus* **	*Typha* spp., *Peltandra virginica*, *I. brevicaulis +*, *I. cristata +*, *I. lacustris +*, *I. robusta* [*versicolor* × *virginica*] *+*, *I. setosa*, *I. verna +*, *I. versicolor*, *I. virginica var. shrevei +*, sedges, rushes
Emergent	*Juncus compressus* *	*Juncus alpinus +*, *J. ambiguous +*, *J. balticus*, *J. biflorus +*, *J. marginatus*, *J. brachycarpus*, *J. brachycephalus +*, *J. dichotomus +*, *J. diffusissimus +*, *J. ensifolius +*, *J. greenei +*, *J. interior +*, *J. militaris +*, *J. pelocarpus +*, *J. scirpoides +*, *J. secundus +*, *J. stygius +*, *J. subcaudatus +*, *J. vaseyi +*, non-rushes
Emergent	*Juncus inflexus* *	*J. alpinus +*, *J. ambiguous +*, *J. balticus*, *J. biflorus +*, *J. marginatus*, *J. brachycarpus*, *J. brachycephalus +*, *J. dichotomus +*, *J. diffusissimus +*, *J. ensifolius +*, *J. greenei +*, *J. interior +*, *J. militaris +*, *J. pelocarpus +*, *J. scirpoides +*, *J. secundus +*, *J. stygius +*, *J. subcaudatus +*, *J. vaseyi +*
Emergent	*Lycopus asper*	*L. americanus*, *L. amplectens +*, *L. rubellus +*, *L. uniflorus*, *L. virginicus +*
Emergent	*Lycopus europaeus*	*L. americanus*, *L. amplectens +*, *L. rubellus +*, *L. uniflorus*, *L. virginicus +*
Emergent	*Lythrum salicaria* **	Grasses, sedges, flowering plants
Emergent	*Epilobium hirsutum* *	*E. angustifolium +*, *E. strictum +*
Emergent	*Echinochloa crus-galli*	*E. muricata*, *E. walteri +*,
Emergent	*Phragmites australis* ssp. *Australis* **	Sedges, rushes, cattails
Emergent	*Lysimachia nummularia* *	*L. radicans +*
Emergent	*Lysimachia vulgaris* *	*L. radicans +*
Emergent	*Typha angustifolia* **	*T. latifolia*, *Campanula aparinoides*, *Cicuta bulbifera*, *Galium tinctorium*
Emergent (tree)	*Frangula alnus* **	*Rhamnus alnifolia +*, *Rhamnus lanceolata +*, *Ilex verticillata +*, trees, shrubs and wildflowers
Emergent (tree)	*Salix purpurea*	Native willows

## Supplementary Materials

The following are available online at https://www.mdpi.com/2223-7747/10/2/406/s1, Profiles and current distribution maps for each species can be found at https://www.glerl.noaa.gov/glansis/ (accessed on 1 September 2020). Detailed impact assessments including noncompetition components of impact are published in the Appendices of NOAA TM-161a–c and available in full text at https://www.glerl.noaa.gov/pubs/#techRep (accessed on 15 January 2020). Original sources for all map data and impact statements can be found in these respective sources.
