# Supplementary material for: A Review and Secondary Analysis of Competition-Related Impacts of Nonindigenous Aquatic Plants in the Laurentian Great Lakes
Source: Plants (Basel). 2021 Feb 20;10(2):406. doi: 10.3390/plants10020406 (PMC7924059; doi:10.3390/plants10020406)
Supplement: Supplementary file 1 [file plants-10-00406-s001.pdf]

**Supplementary Materials:**

Profiles and current distribution maps for each species can be found at https://www.glerl.noaa.gov/glansis/. Detailed impact assessments including noncompetition components of impact are published in the Appendices of NOAA TM-161a–c and available in full text at https://www.glerl.noaa.gov/pubs/#techRep. Original sources for all map data and impact statements can be found in these respective sources.
